# Glioblastoma cells have increased capacity to repair radiation-induced DNA damage after migration to the olfactory bulb

**DOI:** 10.1186/s12935-022-02819-0

**Published:** 2022-12-08

**Authors:** Charlotte Degorre, Ian C. Sutton, Stacey L. Lehman, Uma T. Shankavaram, Kevin Camphausen, Philip J. Tofilon

**Affiliations:** Radiation Oncology Branch, National Cancer Institute, 10 Center Drive-MSC 1002, Building 10, B3B69B, Bethesda, MD 20892 USA

**Keywords:** Radioresistance, Glioblastoma, Microenvironment

## Abstract

**Background:**

The invasive nature of GBM combined with the diversity of brain microenvironments creates the potential for a topographic heterogeneity in GBM radioresponse. Investigating the mechanisms responsible for a microenvironment-induced differential GBM response to radiation may provide insights into the molecules and processes mediating GBM radioresistance.

**Methods:**

Using a model system in which human GBM stem-like cells implanted into the right striatum of nude mice migrate throughout the right hemisphere (RH) to the olfactory bulb (OB), the radiation-induced DNA damage response was evaluated in each location according to γH2AX and 53BP1 foci and cell cycle phase distribution as determined by flow cytometry and immunohistochemistry. RNAseq was used to compare transcriptomes of tumor cells growing in the OB and the RH. Protein expression and neuron–tumor interaction were defined by immunohistochemistry and confocal microscopy.

**Results:**

After irradiation, there was a more rapid dispersal of γH2AX and 53BP1 foci in the OB versus in the RH, indicative of increased double strand break repair capacity in the OB and consistent with the OB providing a radioprotective niche. With respect to the cell cycle, by 6 h after irradiation there was a significant loss of mitotic tumor cells in both locations suggesting a similar activation of the G2/M checkpoint. However, by 24 h post-irradiation there was an accumulation of G2 phase cells in the OB, which continued out to at least 96 h. Transcriptome analysis showed that tumor cells in the OB had higher expression levels of DNA repair genes involved in non-homologous end joining and genes related to the spindle assembly checkpoint. Tumor cells in the OB were also found to have an increased frequency of soma–soma contact with neurons.

**Conclusion:**

GBM cells that have migrated to the OB have an increased capacity to repair radiation-induced double strand breaks and altered cell cycle regulation. These results correspond to an upregulation of genes involved in DNA damage repair and cell cycle control. Because the murine OB provides a source of radioresistant tumor cells not evident in other experimental systems, it may serve as a model for investigating the mechanisms mediating GBM radioresistance.

**Supplementary Information:**

The online version contains supplementary material available at 10.1186/s12935-022-02819-0.

## Background

Glioblastoma (GBM) is the most frequent primary adult brain tumor with radiotherapy serving as a major treatment modality [1]. Whereas radiotherapy prolongs patient survival, even in combination with surgery and chemotherapy, the median survival of GBM patients remains below 2 years with the 5-year survival rate under 10% [1]. Because increased total radiation dose fails to improve local control and recurrences are predominantly within the initial treatment volume, GBMs have long been considered radioresistant. Towards improving GBM therapy, the standard approach aspires to define the mechanisms mediating its radioresistance with the subsequent design of target-based strategies for enhancing GBM cellular radiosensitivity. However, a major barrier in this process is the availability of an experimental model that replicates GBM radioresistance. The in vitro radiosensitivity of long-established human glioma cell lines, which have little in common with the biology of GBMs in situ [2], is not significantly different from that of cell lines initiated from tumors that typically respond to radiotherapy [3]. Stem-like cells isolated from GBMs (referred to as GSCs) simulate GBM biology and are considered to play a critical role in initiating and maintaining GBMs. Yet, with respect to in vitro radiosensitivity, GSCs are significantly more sensitive than established glioma lines [4].

The uncertainty of in vitro models as representative of GBM radioresistance suggested that in vivo conditions need to be considered. Direct comparisons of radiation-induced nuclear foci in GBM cells growing in vitro to those growing as orthotopic tumors implied that the cells irradiated in vivo were significantly more radioresistant [5]. These lab results were consistent with clinical observations indicating that, although comprising a diverse set of tumors displaying extensive biological heterogeneity, all GBMs essentially fail radiotherapy. This relatively homogeneous clinical response in a background of substantial intertumor heterogeneity further implicates the brain environment as a determinant of radioresistance. However, the brain is comprised of a variety of distinct microenvironments and GBMs are highly invasive creating the potential for a topographical influence on GBM radioresponse.

To address this form of intratumor heterogeneity we previously used brain tumors initiated from GSCs implanted into the right striatum of nude mice with radiosensitivity evaluated at the individual cell level using incorporation of a halogenated thymidine analog to identify proliferating cells [6]. In this model, the GSCs (NSC11 and NSC20) implanted into the right striatum invade extensively throughout the right hemisphere (RH), including into the olfactory bulb (OB). After irradiation, the number of proliferating cells in the RH, which included the striatum, corpus callosum and cortex, remained significantly below control levels out to at least 20 days, while in the OB they began to recover at 4 days and returned to control levels by 12 days, indicating that tumor cells in the OB are relatively radioresistant. These results are supported by a separate study in which NSC11 and NSC20 brain tumors from control and irradiated mice were collected at morbidity and their growth patterns compared [7]. Whereas tumor cells in unirradiated mice were diffusely distributed throughout most of the right hemisphere, in irradiated mice the tumors were less infiltrative with cells primarily limited to the anterior portion of the hemisphere and the OB. Because the murine OB provides a source of radioresistant tumor cells not evident in other experimental systems, it may serve as a model for investigating the mechanisms mediating GBM radioresistance. In the study presented here the radiation-induced DNA damage response was compared between tumor cells growing in the OB with those in the RH. Data shows that as compared to GSCs growing in the RH, those growing in the OB have an increased capacity to repair radiation induced DSBs and a prolonged accumulation in G2. Transcriptome analysis suggests that these differences can be attributed to changes in GSCs gene expression within the OB. Finally, the frequency of somatic contacts between tumor cells and neurons were significantly greater in the OB.

## Materials and methods

### Glioblastoma stem-like cell lines

GSC lines NSC11 and NSC20 (provided by Dr. Frederick Lang, MD Anderson Cancer Center in 2008 as frozen stocks) were grown as neurospheres in stem cell medium consisting of DMEM/F-12 (Invitrogen), B27 supplement (Invitrogen), and human recombinant bFGF and EGF (50 ng/ml each, R&D Systems) at 5%CO_2_/5%O_2_ and 37 °C. For use in experiment CD133+ GSC cells were isolated by FACS as reported previously [4] and maintained in neurosphere culture. Each cell line was cultured less than 2 months after resuscitation; tested negative for mycoplasma by PCR and authenticated by routine morphologic and growth analysis. NSC11 and NSC20 cells were transduced with a lentivirus containing the Green Fluorescent Protein (eGFP2) and the bioluminescent Luciferase enzyme (ffLuc2) under the UbC promoter control (LVpFUGQ-UbC-ffLuc2-eGFP2) [8].

### Xenografts

CD133+ GSCs (1.0 × 10^5^) transduced to express luciferase and GFP were implanted into the right striatum of 6-week-old athymic female nude mice (NCr nu/nu; NCI Animal Production Program) [5]. Bioluminescent imaging (BLI) was performed as described [7]. For survival analyses, on day 21 after implantation mice were randomized according to BLI signal into two groups: control (11 and 7 mice for NSC11 and NSC20 respectively) and irradiated (10 Gy) (12 and 7 mice for NSC11 and NSC20 respectively). Prior to irradiation, mice were anesthetized by a ketamine/xylazine cocktail and placed in a well-ventilated plexiglass jig with shielding for the entire torso and critical normal structures of the head (ears, eyes and neck) with radiation delivered using an X-Rad 320 X-irradiator (Precision X-Rays, Inc.) at a dose rate of 2.9 Gy/minute. Mice were monitored daily until the onset of neurologic symptoms (morbidity) and BLI performed weekly after irradiation until the first mouse of the group was lost. For other experiments, at 40 days post-implantation mice (3–4 mice per group) were irradiated and euthanized at the specified time points. All experiments were performed as approved according to the principles and procedures in the NIH Guide for Care and Use of Animals and were conducted in accordance with the Institutional Animal Care and Use Committee.

### Immunohistochemistry

After perfusion via cardiac puncture with chilled PBS followed by 10% buffered formalin, brains were removed, placed in 10% buffered formalin before sectioning and embedded in paraffin. For all studies, sagittal sections of the RH, which included the cortex, striatum and white matter tracts, and coronal sections of the corresponding OB were evaluated. For analyses of γH2AX and 53BP1, paraffin embedded brains were cut into 10 μm thick sections, deparaffinized in xylene, rehydrated in decreasing grades of alcohol followed by heat-induced antigen retrieval in citrate solution (pH 6). Slides were incubated with primary antibodies to γH2AX (Millipore, 05-636) or 53BP1 (Cell Signaling, 4937) in PBS containing 10%FBS, 1%BSA and 0.3% Triton-X-100 overnight at 4 °C along with antibody to Sox2 (Cell signaling #3579 for γH2AX or LSBio C761895 for 53BP1), followed by Alexa fluor conjugated secondary antibodies from Invitrogen (Alexa fluor-555 for Sox2, Alexa fluor-647 for γH2AX and 53BP1) with 1µg/ml DAPI for 1 h at room temperature and mounted with Prolong Diamond antifade (Invitrogen). Imaging was performed using the Carl Zeiss LSM 780 laser scanning confocal using the objectives 40x with oil. To determine the number of γH2AX and 53BP1 foci per nucleus, orthogonal projections of confocal image stacks were done using the Zen 2.3 software. The number of foci per nucleus was then determined in a minimum of 100 cells per mouse at each location. Corrected Total Cell Fluorescence (CTCF) was calculated as: integrated density minus (area of selected cell x mean fluorescence of background) using ImageJ and was used as a measure of fluorescence intensity. Analysis of additional proteins used 6 to 8 μm sections and followed the same basic procedures. The antibodies used are listed in Additional file 1: Supplemental Methods. Imaging was performed using the Epifluorescence Zeiss microscope for the GABA and TH; confocal microscopy was used for the MAP2.

### Flow cytometry

At designated times after irradiation, mice were euthanized, and cardiac perfusion was performed with cold PBS. Tumor tissue was separated from OB (2 OBs per sample) and RH based on GFP expression as viewed under the stereoscope, cut into ~ 1 mm fragments and incubated in accutase (Stempro) at 37 °C for 30 min followed by dilution in PBS and filtration through a 40 μm cell strainer. After a centrifugation (10 min, 1500 rpm), red blood cells were removed using the RBC lysis solution (MACS 130-094-183) according to the manufacturer protocol. For cell cycle analysis, cells were fixed in 70% cold ethanol and stored overnight at − 20 °C. After washing with PBS, cells were stained with 10 µg/ml propidium iodide (PI) containing 100ug/ml RNAseA (Fermantas EN0531) and analyzed by flow cytometry (Fortessa BD bioscience). In this analysis gating was done on GFP positive cells, allowing the exclusion of normal mouse cells and the quantification of tumor cells only (Additional file 2: Fig. S1 FCM).

### RNAseq

For transcriptome analysis, mice were euthanized with brains collected after PBS cardiac perfusion. GFP expressing tissue (tumor) was separated from the OB and RH under the stereoscope and flash frozen. Two OBs and one RH per sample were used to obtain sufficient material. RNA was extracted from the tissue with the Allprep DNA/RNA/protein mini kit (Qiagen) according to the manufacturer protocol. RNA-seq was performed on the purified RNA by the Center for Cancer Research Sequencing Facility in Frederick, Maryland. Briefly, 200 ng of RNA was used as input for mRNA capture with oligo-dT coated magnetic beads. The mRNA was fragmented, followed by random-primed cDNA synthesis. The resulting double-stranded cDNA was used as the input to a standard Illumina library prep with end-repair, adapter ligation and PCR amplification to generate a sequencing ready library. The final library was then quantitated by qPCR before cluster generation and sequencing on the Illumina Nextseq sequencer. All RNAseq NGS data processing occurred using the RNAseq pipelines implemented in the CCBR Pipeliner framework (https://github.com/CCBR/Pipeliner) with adapter sequences removed using Trimmomatic v0.36 [9]. To eliminate mouse reads, FASTQ files were aligned to mouse GRCm38 and human h38 genomes using STAR aligner [10] followed by filtering out reads that were mapped to the mouse genome using bamcmp software [11]. RSEM [12] was then used for gene-level expression quantification, and data processing was performed in R statistical program environment. Data was normalized by trimmed mean of M-values (TMM) implemented in edgeR software [13]. Differential gene expression was calculated from sequences mapped to the hg38 genome in edgeR using the GLM approach and likelihood ratio tests. The data discussed in this publication have been deposited in NCBI’s Gene Expression Omnibus [14] and are accessible through GEO Series accession number GSE212161 (https://www.ncbi.nlm.nih.gov/geo/query/acc.cgi?acc=GSE212161). Genes with an adjusted p-value < 0.05 were submitted for Ingenuity Pathway Analysis (IPA) (QIAGEN). Genes were also ranked by (sign of the fold change)*-log_10_(p-value) and submitted to GSEA [15] for pre-ranked analysis with 1000 permutations against the GO database. Gene survival association analysis was performed using the Glioblastoma Bio Discovery Portal (GBM-BioDP) (https://gbm-biodp.nci.nih.gov) [16], which uses data downloaded from the TCGA data portal (https://tcga-data.nci.nih.gov/) [17]. Using GBM-BioDP, currated lists of genes enriched in OB were used to generate the Kaplan-Meier curves and compared to overall survival of GBM patients stratified by prognostic index, which was computed by weight averaging the gene expressions with the regression coefficients of a multi-gene Cox proportional hazards model.

### Statistics

Statistical significance was determined using a two-tailed Student’s t-test. For in vivo survival studies, Kaplan-Meier curves were generated, and log-rank values calculated in GraphPad Prism 9 (GraphPad Software).

## Results

### Radiation-induced DNA damage and repair

**Fig. 1:**
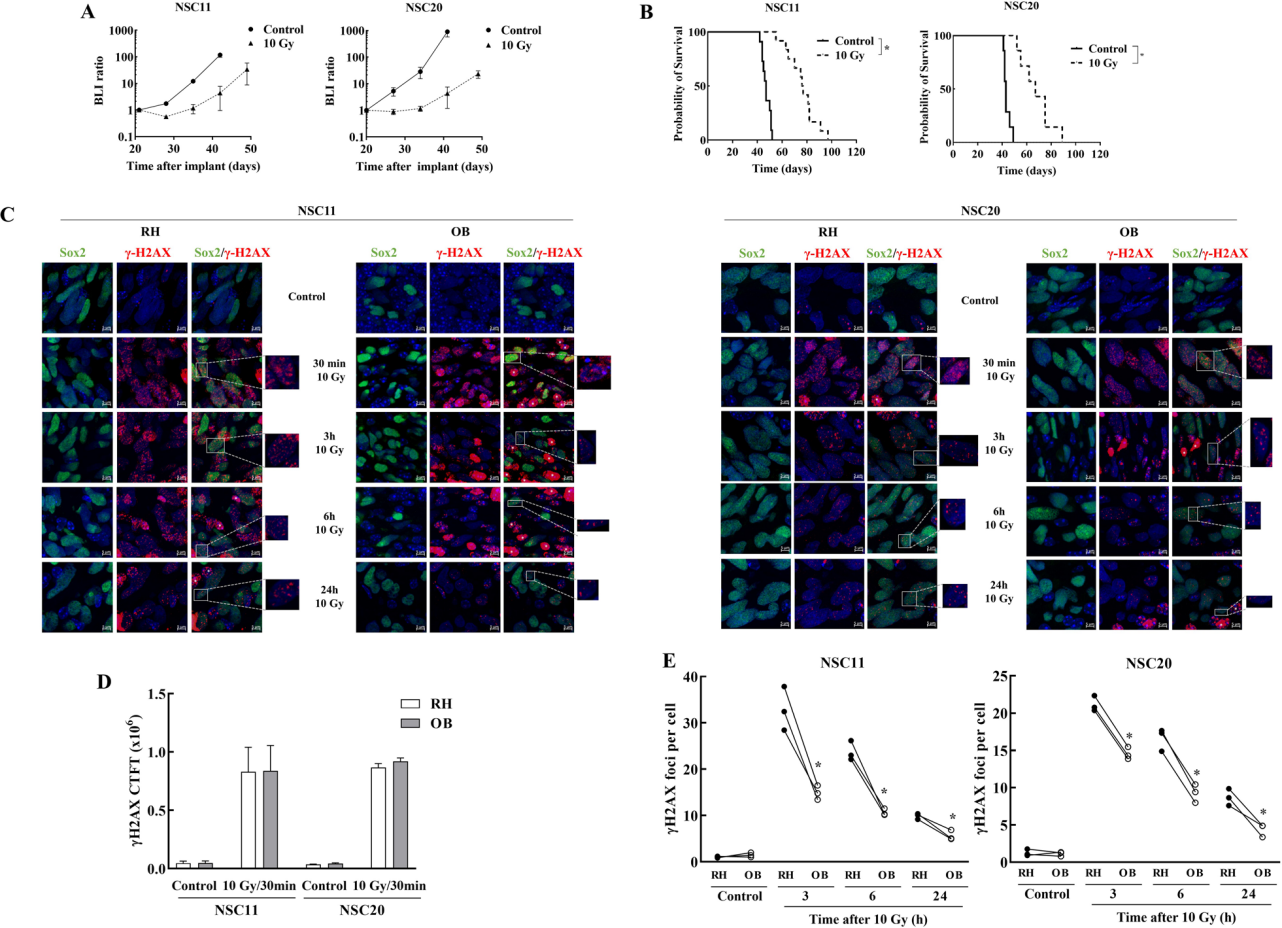
γH2AX analysis.  **A**, **B** Tumor response to 10 Gy delivered on day 21 post-implant. **A** BLI total flux ratio as a function of time after irradiation of NSC11 and NSC20 tumors. Values represent the mean ± SEM. **B** Kaplan–Meier survival curves generated for NSC11 and NSC20 tumor-bearing mice with log-rank analysis (*p < 0.0001). For γH2AX analyses 10 Gy was delivered to mice bearing NSC11 or NSC20 tumors 40 days post-implant. **C** Representative matched confocal images of γH2AX in NSC11 and NSC20 cells in OB and RH as a function of time after 10 Gy (×40 magnification—orthogonal projection from confocal images, DAPI, Sox2 and γH2AX are shown in blue, green and red, respectively) with mouse cells indicated by the white asteriscs. **D** γH2AX expression was determined by fluorescence intensity (CTCF) at 30 min after 10 Gy in NSC11 and NSC20 tumor cells in OB and RH. Values represent the mean ± SEM, n = 3. **E** γH2AX foci in NSC11 and NSC20 tumor cells as a function of time after 10 Gy in OB and RH. Each point corresponds to an individual mouse with at least 100 cells analyzed in each location with lines connecting the matched OB (white circle) and RH (black circle) from the same mouse. *p-val < 0.05 refers to the mean ± SEM of OB versus RH

To investigate the DNA damage response in individual tumor cells located in the OB and RH, mice bearing brain tumors initiated from GSCs were irradiated with a single dose of 10 Gy. Delivery of 10 Gy to mice with NSC11 or NSC20 brain tumors delays tumor growth as measured by BLI and prolongs the animal survival, indicative of a significant anti-tumor effect (Fig. 1A and B). Moreover, as shown below and in contrast to the clinically relevant dose of 2 Gy [5], 10 Gy was sufficient to induce detectable levels of DNA damage. To evaluate the DNA damage response after 10 Gy, initial studies addressed γH2AX foci, which reflect DNA double strand breaks (DSBs), the lethal event mediating radiation-induced cell death. For this experiment, 40 days after implantation of tumor cells into the right striatum, mice were irradiated (10 Gy) and brains collected at specified times. γH2AX analysis was performed on sagittal sections of the right hemisphere, which includes the corpus callosum and striatum, and on coronal sections of the OB, each co-stained for Sox2 to identify tumor cells. Representative micrographs for NSC11 and NSC20 tumors are shown in Fig. 1C. Because the large number of foci induced at 30 min post 10 Gy in both locations prevented accurate quantification of foci number, fluorescence intensity (Corrected Total Cell Fluorescence, CTCF) was used as a measure of γH2AX expression. As shown in Fig. 1D, similar levels of γH2AX expression were detected in the OB and RH at 30 min after 10 Gy suggesting similar levels of initial damage for both NSC11 and NSC20 GSCs. In contrast to 30 min, γH2AX foci in tumor cells in the OB and RH at 3-24 h after irradiation could be accurately counted (Fig. 1E); γH2AX foci per tumor cell declined in both locations in a time dependent manner, consistent with the repair of DSBs. However, at each time point after 10 Gy the number of γH2AX foci were significantly lower in the OB versus the RH. For both GSC-initiated tumors, at 24 h after 10 Gy the γH2AX foci remaining in tumor cells in the OB were significantly closer to unirradiated levels than those in the RH. These results suggest that while similar levels of DSBs are induced in the OB and RH, tumor cells in the OB have an increased repair capacity.

**Fig. 2:**
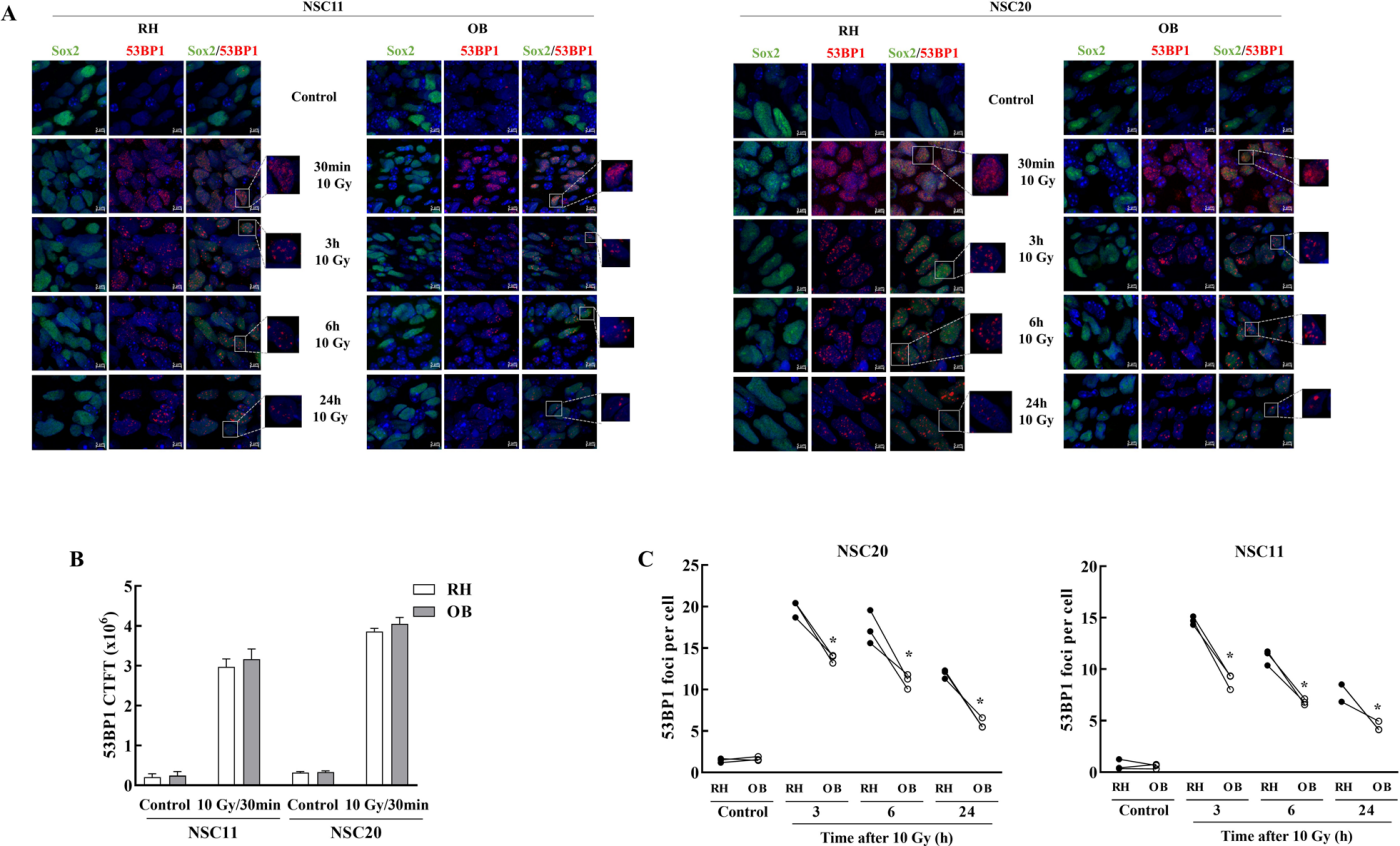
53BP1 analysis. For 53BP1 analyses 10 Gy was delivered to mice bearing NSC11 or NSC20 tumors 40 days post-implant. **A** Representative matched confocal images of 53BP1 in NSC11 and NSC20 tumor cells in OB and RH as a function of time after 10 Gy (×40 magnification—orthogonal projection from confocal images, DAPI, Sox2 and 53BP1 are shown in blue, green and red, respectively). **B** 53BP1 expression was determined by fluorescence intensity (CTCF) at 30 min after 10 Gy in NSC11 and NSC20 tumor cells in OB and RH. Values represent the mean ± SEM, n = 3. **C** 53BP1 foci in NSC11 and NSC20 tumor cells as a function of time after 10 Gy in OB and RH. Each point corresponds to an individual mouse with at least 100 cells analyzed in each location with lines connecting the matched OB (white circle) and RH (black circle) from the same mouse. At 24 h after 10 Gy (n = 3), the number of foci per cells were similar between the biological replicates and consequently only two lines are visible on the graphs. *p-val < 0.05 refers to the mean ± SEM of OB versus RH

53BP1, a critical protein participating in the radiation-induced DNA damage response, also serves as a marker of DSBs and their repair [18]. Representative images of 53BP1 foci in NSC11 and NSC20 cells within the OB and RH are shown in Fig. 2A. As for γH2AX, due to the large number of foci at 30 min after 10 Gy, fluorescent intensity was used to measure 53BP1 expression. As shown in Fig. 2B, similar levels of 53BP1 expression were detected in tumor cells in the OB and RH at 30 min after 10 Gy. In contrast to 30 min, 53BP1 foci in tumor cells in the OB and RH at 3–24 h after irradiation could be accurately counted; foci per tumor cell declined in both locations in a time dependent manner, consistent with the repair of DSBs. However, at each time point after 10 Gy the number of 53BP1 foci were significantly lower in the OB versus the RH (Fig. 2C). For both GSC-initiated tumors, at 24 h after 10 Gy the 53BP1 foci remaining in tumor cells in the OB were significantly closer to unirradiated levels than those in the RH. As for γH2AX (Fig. 1), the 53BP1 results suggest that tumor cells growing in the OB have an enhanced capacity to repair radiation-induced DSBs.

### Cell cycle phase redistribution after 10 Gy

**Fig. 3:**
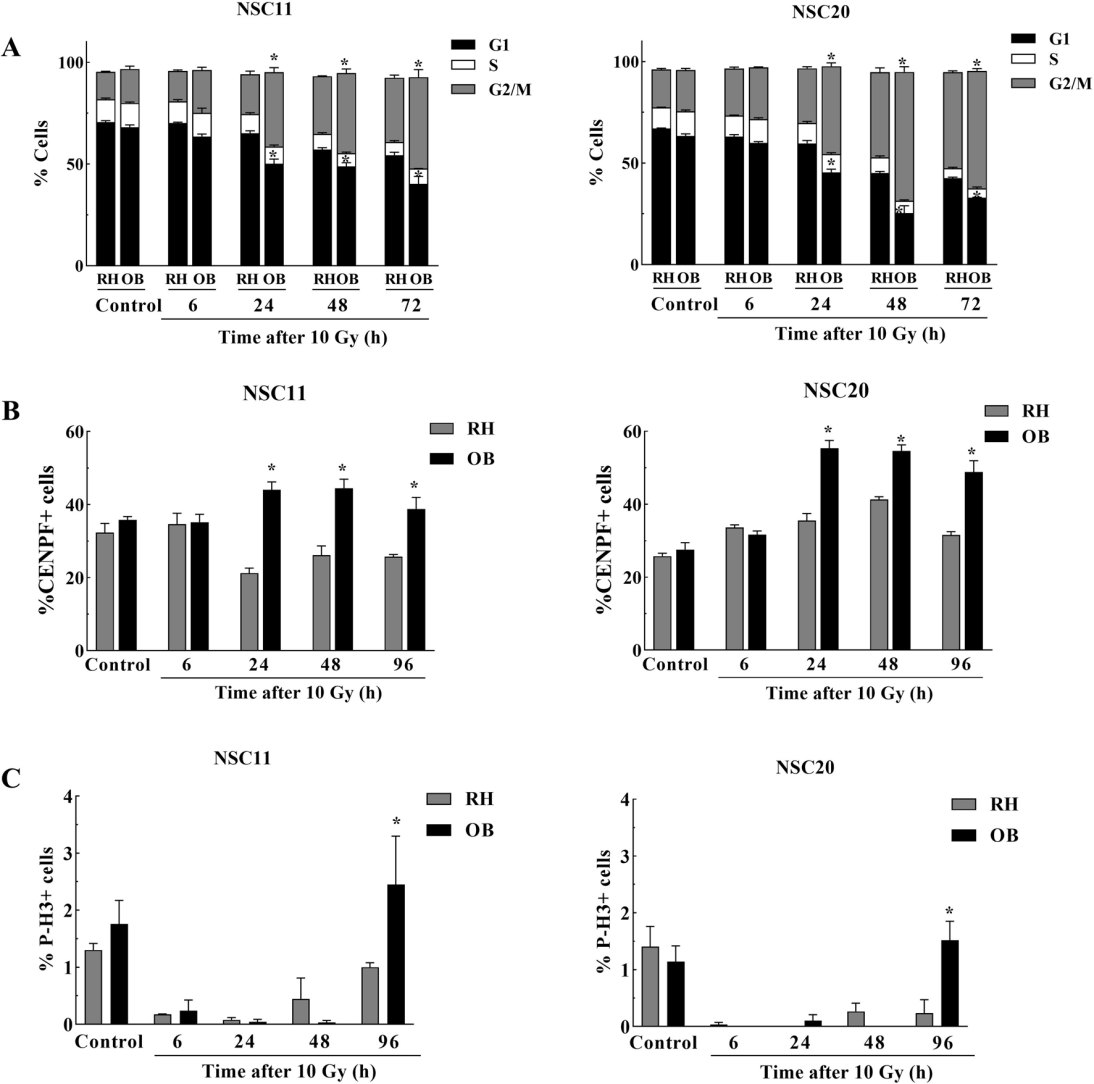
Cell cycle phase distribution. For cell cycle phase analyses 10 Gy was delivered to mice bearing NSC11 or NSC20 tumors 40 days post-implant with tumors collected at specified time points. **A** Cell cycle phase distribution of GFP expressing cells as defined using flow cytometry. Bars represent the percentage of NSC11 and NSC20 GSCs in each cell cycle phase in OB and RH. Distributions represent the mean ± SEM for 3–4 mice. **B** Immunohistochemical determination of the percentage of tumor cells expressing the G2 marker CENPF with tumor cells identified by Sox2 staining. Values represent the mean ± SEM for 3–4 mice. **C** Immunohistochemical determination of the percent tumor cells expressing the mitosis marker phospho-H3 with tumor cells identified by Sox2 staining. Values represent the mean ± SEM for 3–4 mice. *p-val < 0.05

Activation of cell cycle checkpoints is a critical component of the radiation-induced DDR. Accordingly, tumor tissue was isolated from the right OB and RH, disaggregated into single cells and flow cytometry used to define cell cycle phase distribution of GFP expressing cells (Fig. 3A and Additional file 2: Fig. S1). For both NSC11 and NSC20 tumors, no differences in cell cycle phase distribution were detected between OB and RH tumor cells collected from unirradiated mice or from those at 6 h after 10 Gy. However, 24-72 h post-irradiation, the percent of tumor cells in G2/M increased and in G1 decreased in the OB as compared to the RH. As an alternative method for G2 phase analysis and to avoid tissue disaggregation, levels of the G2 specific protein CENPF were determined with Sox2 co-staining to identify tumor cells. For both NSC11 and NSC20 tumors, no differences in CENPF expression were detected between OB and RH in unirradiated mice or at 6 h after 10 Gy (Fig. 3B). At 24 to 96 h post-10 Gy the percentage of CENPF positive tumor cells in the OB was significantly greater than in RH indicating an increase in G2 cells in OB as compared to those in the RH by 24 h after irradiation. Data generated using both methods thus suggest that radiation-induced cell cycle phase distribution is influenced by the microenvironment with a greater accumulation of tumor cells into G2 in the OB.

To quantitate cells in mitosis, sections were immuno-stained for phospho-H3 and Sox2. The percentage of mitotic cells in unirradiated tumors were similar in the OB and RH for both NSC11 and NSC20 tumors (Fig. 3C). By 6 h after 10 Gy, there was a significant decrease of the phospho-H3 positive NSC11 and NSC20 tumor cells in both locations, consistent with the initial activation of the G2/M checkpoint. This initial loss of mitotic cells was maintained out to at least 48 h; at 96 h an increase in mitotic cells in the OB as compared to the RH was detected. Of note, the return of mitotic tumor cells to control levels in the OB by 96 h is consistent with previous results showing a return of proliferating cells defined by CldU incorporation in the OB and not in the RH [6].

### Transcriptome analysis of tumor cells in the OB versus RH

**Fig. 4:**
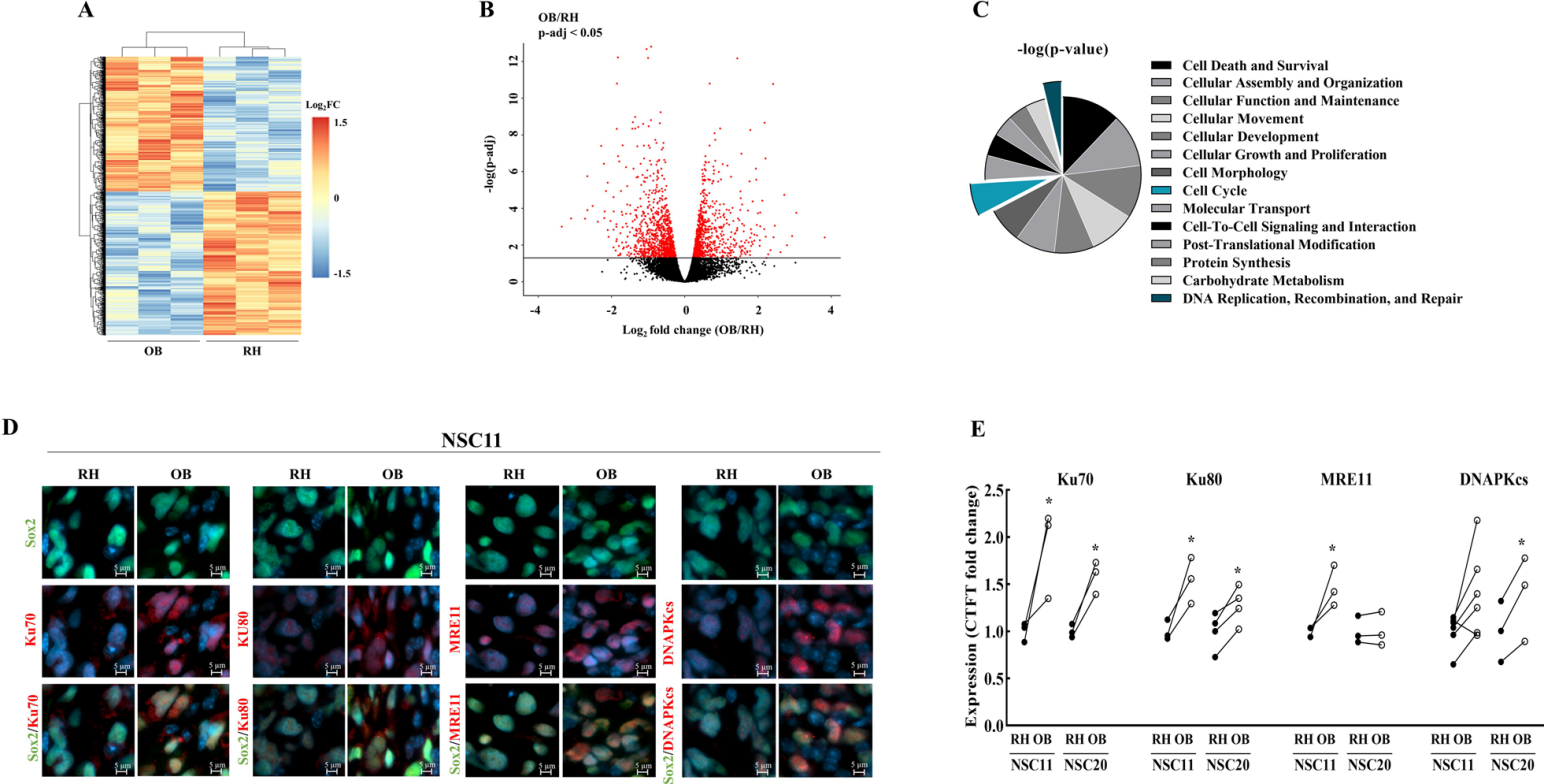
Gene expression analysis of NSC11 cells located in the OB versus RH. On day 40 post-implant, RNA was extracted from NSC11 tumor tissue located in OB and RH and analyzed by RNAseq. **A** Heat map comparing OB and RH samples (3 samples per group). **B** Volcano plot of transcripts significantly (red) changed in OB compared to RH. **C** IPA defined molecular and cellular functions differentially expressed in NSC11 cells in OB versus the RH (all had p-values < 3.40E−09). **D** Representative matched images of NHEJ proteins expression in NSC11 tumor cells in OB vs. RH (DAPI, Sox2 and proteins of interest are shown in blue, green and red, respectively). **E** NHEJ proteins expression was determined by fluorescent intensity (CTCF fold change) in NSC11 and NSC20 GSCs in OB vs. RH. Each point corresponds to an individual mouse with at least 100 cells analyzed in each location with lines connecting the matched OB (white circle) and RH (black circle) from the same mouse. Values represent the mean ± SEM, n = 3–4. *p-val < 0.05

To further investigate the mechanisms mediating the radioresistance of GBM cells in the OB as compared to the RH, RNA-seq was performed on tumor tissue located in each location with contaminating mouse reads filtered out as described by Khandelwal et al. [11]. Specifically, 40 days after implantation of NSC11 cells into the right striatum, tumor tissue (GFP+) was isolated from the right OB and RH, RNA extracted and subjected to RNA-seq based gene expression analysis. To obtain sufficient mRNA for analysis, whereas a single RH from a tumor bearing mouse was sufficient, it was necessary to combine the right OB from 2 tumor bearing mice. As shown in Fig. 4A, clear and consistent differences in transcriptomes were detected between the 3 OB samples and the 3 RH samples. With respect to individual genes, 1015 were increased and 872 decreased in tumor cells in the OB as compared to the RH (Fig. 4B). The functional significance of these changes was evaluated using IPA (Fig. 4C). Among the top molecular cellular functions of the affected genes was *DNA Replication, Recombination, and Repair*: processes involved in the regulation of radiosensitivity. Specifically, 4 genes critical to the repair of DNA DSBs (*MRE11*, *XRCC5*, *XRCC6* and *PRKDC*) were overexpressed in the OB versus the RH. To determine whether the RNAseq results extend to the protein level, immunohistochemical analysis of the corresponding proteins was performed using a cohort of mice independent of those used for RNAseq analysis (Fig. 4D). In addition, to determine whether putative differences were unique to NSC11 tumors, NSC20 tumors were also evaluated. In each mouse and for both NSC11 and NSC20 cells, Ku70 (*XRCC5*) and Ku80 (*XRCC6*) were detected at higher levels in the OB than in the RH (Fig. 4E). Mre11 was expressed at higher levels in the OB for NSC11 tumors, but not in NSC20. DNAPKcs (*PRKDC*) was overexpressed in the OB of NSC20 tumors in all 3 mice evaluated; in NSC11 tumors, DNAPKcs was elevated in the OB in 5 of 6 tumors. For DNAPKcs, of the first 3 mice evaluated, 2 showed increased levels in the OB; one showed a relative increase in the RH. In the second group of mice, all 3 showed an increase in the OB as compared to the RH. In general, these data, consistent with the RNAseq results, indicate that the expression of proteins critical to the repair of DNA DSBs, specifically by the NHEJ pathway, are overexpressed in the OB.

**Fig. 5:**
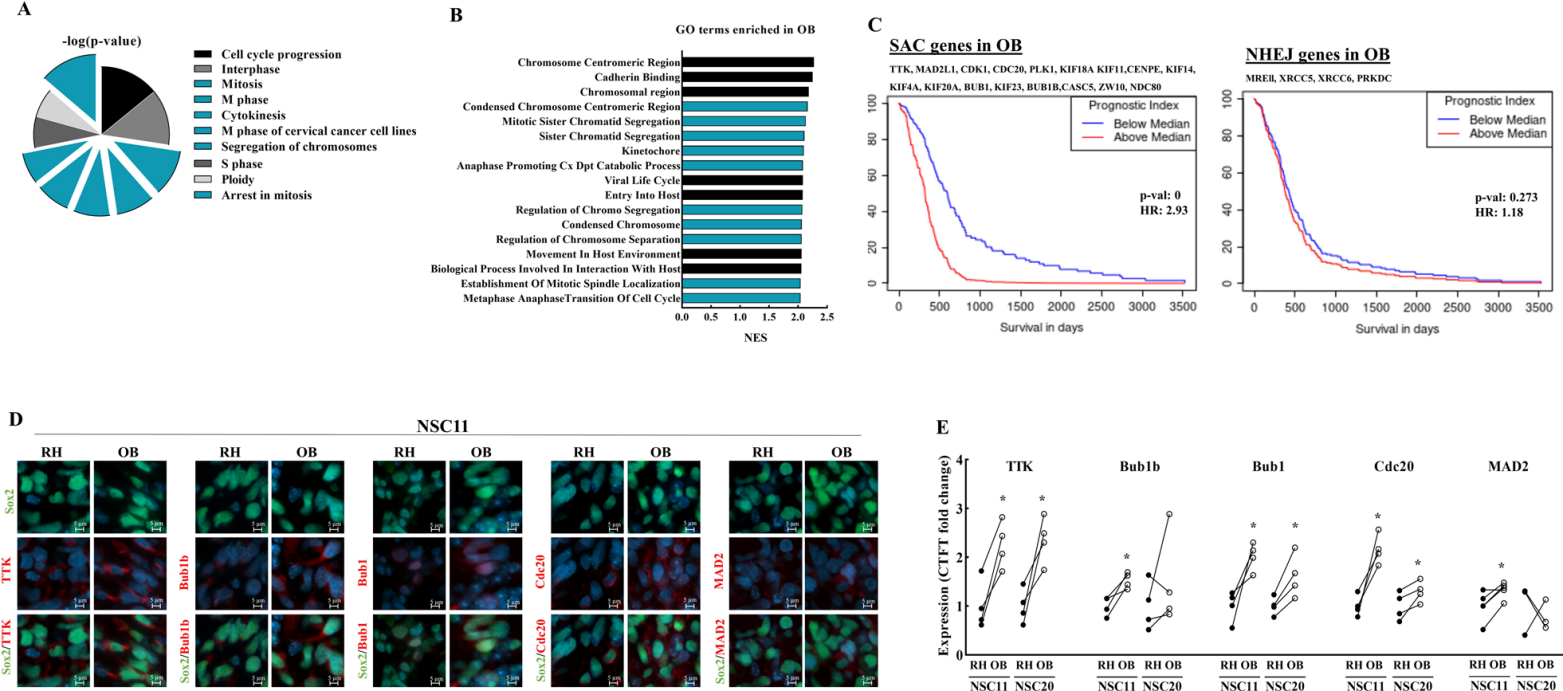
SAC related gene expression in tumor cells. **A** IPA defined subfunctions of the *Cell cycle* molecular function enriched in OB versus RH (all had p-values < 6.05E–08). Highlighted in blue are categories related to *Mitosis*. **B** Top GO terms in OB versus RH as defined by Normalized Enrichment Score (NES). **C** Kaplan–Meier analyses comparing overall survival of GBM patients versus expression of SAC and NHEJ related genes overexpressed in OB. **D** Representative matched images of SAC related proteins in NSC11 and NSC20 tumor cells in OB and RH. (DAPI, Sox2 and proteins of interest are shown in blue, green and red, respectively). **E** SAC protein expression was determined by fluorescent intensity (CTCF fold change) in NSC11 and NSC20 tumor cells in OB vs. RH. Each point corresponds to an individual mouse with at least 100 cells analyzed in each location with lines connecting the matched OB (white circle) and RH (black circle) from the same mouse. Values represent the mean ± SEM, n = 3–5. *p-val < 0.05

Genes assigned to the cellular and molecular function *Cell Cycle*, which plays a role in determining radiosensitivity, were also significantly enriched in the OB versus the RH (Fig. 4C). Further analysis of this category showed that 6 of the top 10 subfunctions were associated with mitosis, including *arrest in mitosis* (Fig. 5A). When the differentially expressed genes in the OB and RH were evaluated using GSEA, 10 of the top 17 GO terms enriched in the OB involved functions related to mitosis (Fig. 5B). The mitosis related genes over-expressed in the OB can more specifically be assigned to core components and regulators of the Spindle Assembly Checkpoint (SAC). A list of the SAC related genes overexpressed in the OB was evaluated in terms of its relationship to GBM clinical response as defined by the TCGA [16]. As shown in Fig. 5C, patients whose tumor expressed high levels of these SAC genes had reduced survivals. The genes over expressed in the OB related to NHEJ, in contrast, showed no correlation with GBM patient survival. To extend the results relating to the SAC associated genes to the protein level, 5 corresponding proteins were evaluated (Fig. 5D and E). TTK, Bub1 and cdc20 were expressed at higher levels in the OB versus the RH in NSC11 and NSC20 tumors. Whereas the predicted increases of Bub1b and Mad2 were detected in the OB for NSC11 tumor cells, no change in protein levels were detected in NSC20 tumors. The results shown in Figs. 4E and 5E suggest that the differences in DNA repair genes and mitosis associated genes predicted from RNAseq data extended to the protein level in NSC11 tumors, which was generally reproducible (6 of 9 genes) in NSC20 tumors.

**Fig. 6:**
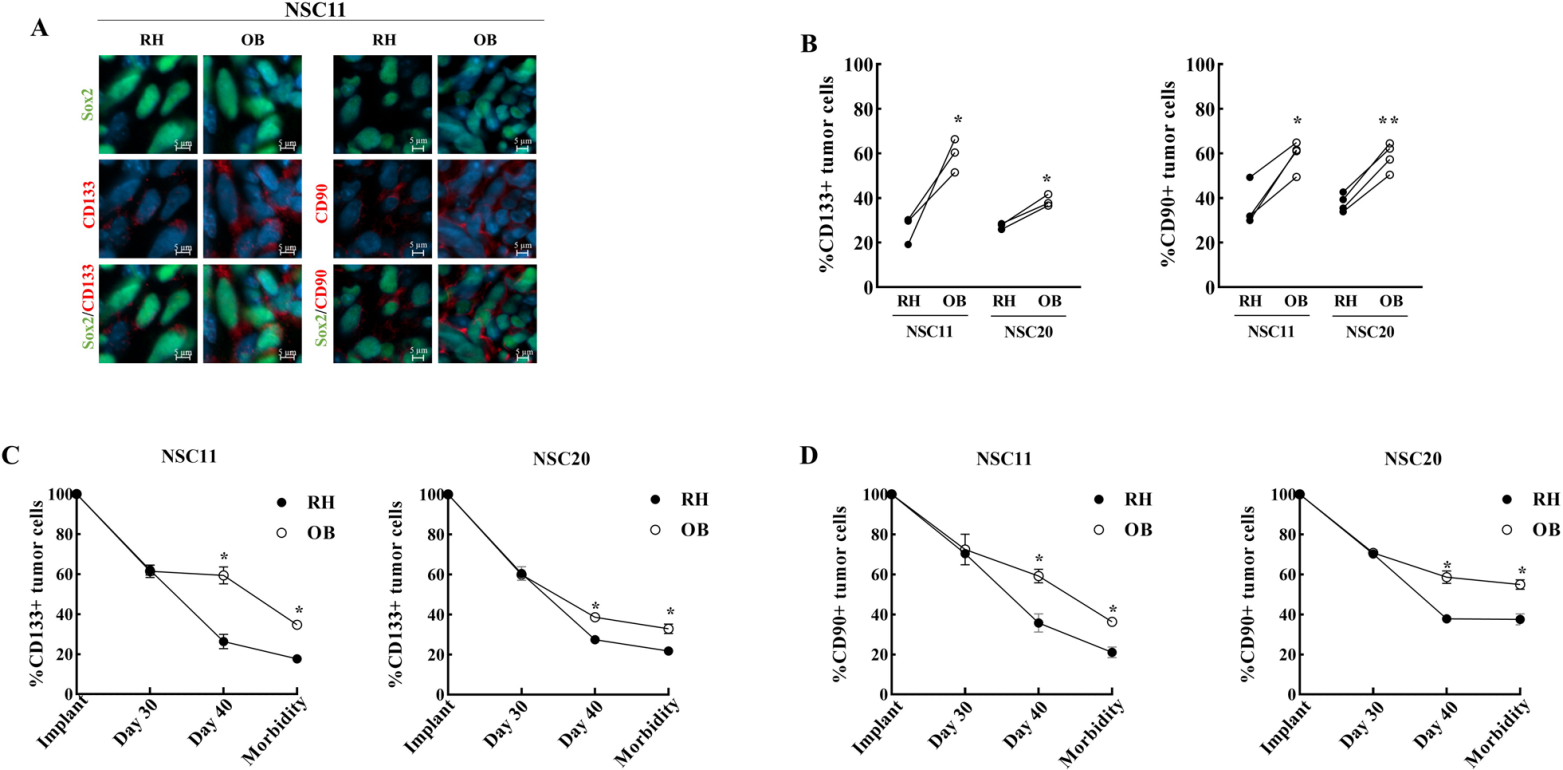
Stem cell marker expression in OB and RH tumor cells. **A** Representative matched images of stem cell marker proteins CD90 and CD133 in NSC11 and NSC20 (Sox2+) tumor cells in OB vs. RH at 40 days post-implant. **B** %CD133+ and CD90+ NSC11 and NSC20 cells in OB and RH. Each point corresponds to an individual mouse with at least 100 cells analyzed in each location with lines connecting the matched OB and RH from the same mouse. **C**, **D** Mice were euthanized at the specified time points after GSC implantation. Matched tumors were analyzed for %CD133+ and CD90+ tumor cells, respectively, as a function of time after GSC implant. Values represent the mean ± SEM, n = 3–4. *p-val < 0.05. The values shown for Day 40 are the same as shown in B above

The genes overexpressed in the OB as compared to the RH for NSC11 tumors included 2 genes that serve as stem-like cell markers (*THY1* and *PROM1*). Immunohistochemical analysis showed an increase in the expression of the corresponding proteins (CD90 and CD133, respectively) in the OB of NSC11 tumors as compared to the RH (Fig. 6A and B). In NSC20 tumors, the expression of CD90 and CD133 were also increased in the OB as compared to the RH. The % of CD133+ and CD90+ cells detected at 40 days post-implant raised the possibility that after implantation into the striatum CD133+/CD90+ GSCs preferentially migrate to the OB. However, as shown in Fig. 6C, D, from the time of implant the %CD133+/CD90+ NSC11 and NSC20 cells in the RH and OB declined to 60–70% at 28 days post-implant with the % positive cells continuing to decrease out to the onset of morbidity. This is consistent with previous results indicating that after implantation GSCs proliferate and differentiate to form a heterogeneous tumor [8]. Although continuing to decrease, a greater percentage of CD133+/CD90 + cells were detected in the OB at 40 days post-implant as well as at the onset of morbidity. These results suggest that CD133+/CD90 + stem-like cells do not preferentially migrate to the OB after implantation, rather the OB microenvironment acts to delay their differentiation.

### Neuron–tumor cell contact

**Fig. 7:**
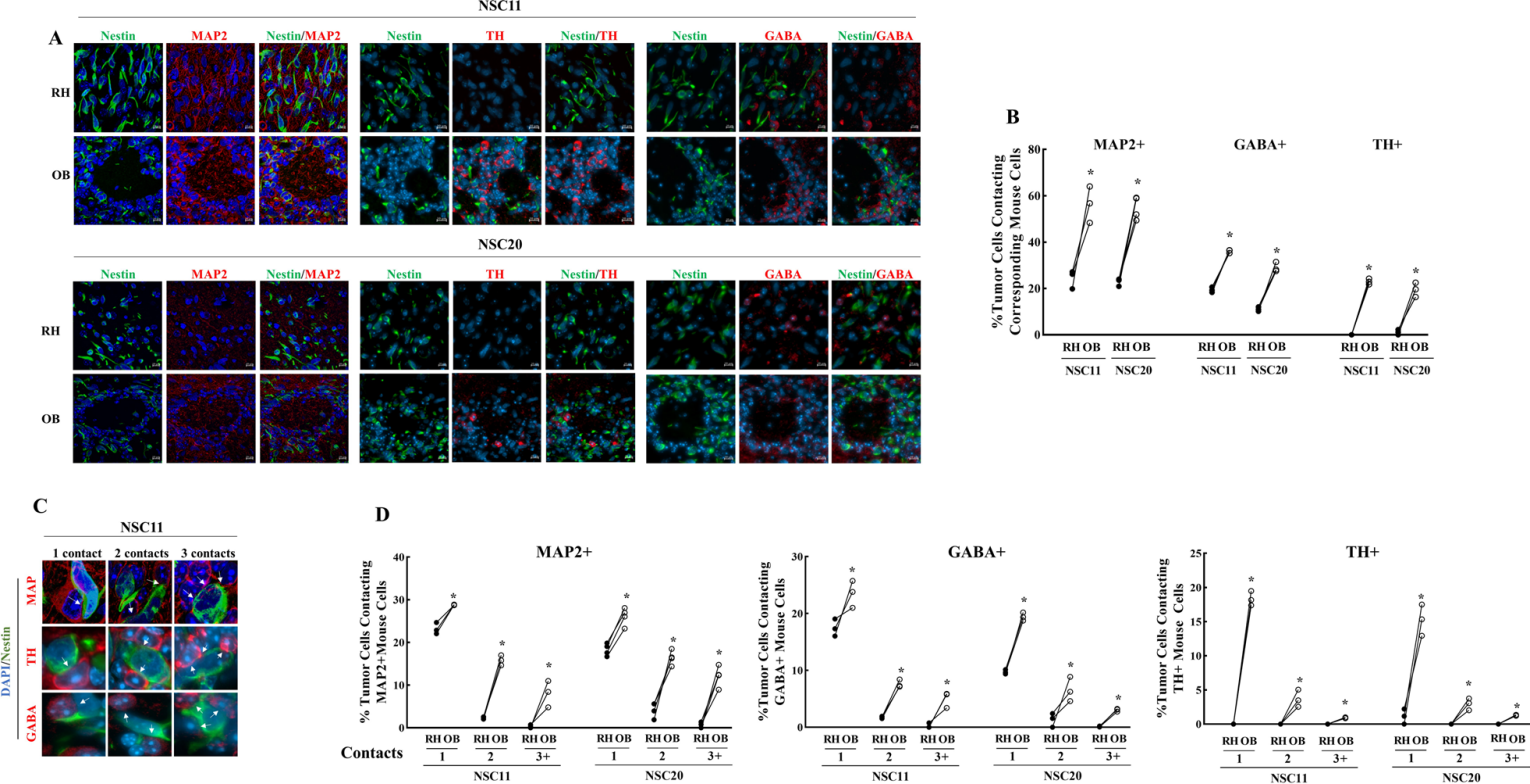
Neuron–tumor cell contact. **A** Representative matched images of NSC11 and NSC20 tumor cells (Nestin+) and MAP2+ neurons, TH+ neurons and GABA+ neurons in the OB and RH (×40 magnification—orthogonal projection from confocal images, DAPI, Nestin and protein of interest are represented in blue, green and red, respectively). **B** %NSC11 and NSC20 cells in contact with each neuron type. Each point corresponds to an individual mouse with at least 100 cells analyzed in each location with lines connecting the matched OB (white circle) and RH (black circle) from the same mouse. **C** Representative zoomed-in images illustrating contact points (white arrows) between NSC11 tumor cells (Nestin+) and MAP2+ neurons, TH+ neurons and GABA+ neurons in the OB. **D** %tumor cells (Nestin+) versus the number of contacts MAP2+ neurons, TH+ neurons and GABA+ neurons. Each point corresponds to an individual mouse with at least 100 cells analyzed in each location with lines connecting the matched OB (white circle) and RH (black circle) from the same mouse. *p-val < 0.05

As an initial investigation into the processes that may account for the differences in tumor cell gene expression in the OB and RH, soma-soma contacts between tumor cells and neurons in the OB and RH were evaluated. Neurons have been suggested to play a role in glioma progression and proliferation [19] and somatic contact between neurons and normal cells in the brain has recently been shown to predict cell–cell interactions and modifications in gene expression [20, 21]. According to the neuron marker MAP2, which identifies all the mature neurons [22, 23], NSC11 and NSC20 cells in the OB had a significantly greater frequency of soma-soma contact with neurons as compared to the tumor cells in the RH (Fig. 7A, B). In the mouse striatum (part of the RH) 90–95% of neurons are GABAergic [24, 25]; the olfactory bulb is comprised of a more diverse set of neurons including dopaminergic and GABAergic [26]. Analysis of NSC11 and NSC20 cell contact with TH+ (dopaminergic) neurons and GABA+ (GABAergic) neurons also revealed significantly greater soma-soma contacts in the OB than in the RH (Fig. 7A, B). Moreover, NSC11 and NSC20 tumor cells in the OB showed a higher number of contacts with MAP2+, TH+ and GABA+ neurons (Fig. 7C and D). These results suggest a greater frequency of cell–cell interactions between neurons and tumor cells in the OB than in the RH.

## Discussion

Consistent with the murine OB providing a radioresistant niche for GBM cells [6], the γH2AX and 53BP1 foci analyses presented here show that, while initial levels of radiation-induced DSBs are similar, tumor cells in the OB have an increased capacity to repair the DNA damage as compared to those in the RH. This appears to be the first report showing that a specific microenvironment within the brain or other organ can differentially modulate DSB repair capacity. Coordinated with DSB repair, activation of cell cycle checkpoints is an additional determinant of radiosensitivity. While radiation activates checkpoints in the G1, S and G2 phases of the cell cycle, it is the rapid activation of the G2/M checkpoint that has consistently been reported to protect against radiation-induced cell death [27]. As shown here, at 6 h after irradiation, in both the OB and RH there was a dramatic loss in M-phase cells suggesting that the G2/M checkpoint is activated in both locations and thus does not play a role in OB mediated radioresistance. However, in the OB there was a greater accumulation of tumor cells in G2 at 24 h after 10 Gy that was maintained out to at least 96 h. Whereas the significance in radiosensitivity is unclear, these results suggest that growth in the OB influences cell cycle regulation after irradiation.

Transcriptome analysis suggests the enhanced DSB repair in tumor cells in the OB can be attributed to an increased expression of a group of genes (MRE11, XRCC5, XRCC6 and PRKDC) essential to the NHEJ pathway of DSB repair, which is a critical determinant of radiosensitivity [28]. MRE11 protein is a component of the MRN complex (MRE11/Rad50/NBS1) playing an essential role in recognizing DSBs and the subsequent actvation of DDR proesses [29]. XRCC5 and XRCC6 genes correspond to Ku80 and Ku70 proteins, respectively, which form heterodimers that bind to radiation-inducd DSB ends and complex with the PRKDC protein product DNA-PKcs. These proteins are critical components of the NHEJ pathway [30]. Thus, the increased expression of these NHEJ genes is consistent with the increased DSB repair capacity detected in the OB as well as the radioresistance of those cells as previously reported [6]. In addition, transcriptome analysis revealed a relative overexpression of genes in the OB corresponding to the components of the SAC, which is typically associated with the accumulation of cells in mitosis. However, cells in mitosis rapidly decreased in both the OB and RH after irradiation, consistent with long established radiobiology indicating that radiation does not activate the SAC. In addition to arrest in mitosis, SAC proteins have also been directly linked to DSB repair. Of the SAC genes overexpressed in the OB tumor cells, *TKK*, *PLK1*, *CDK1*, *BUB1* and *BUB1B* have been shown to play a role in radiosensitivity [28–31]. More recently, members of the kinesin family of motor proteins have been reported to enhance DNA repair, including several overexpressed in the OB (KIF4A, KIF18A, KIF14 and KIF11) [32–34]. Whether the SAC genes overexpressed in the OB play a role in the GBM radiosensitivity individually or as a group reflect a radioresistant phenotype remains to be determined. However, the SAC genes overexpressed in the OB correlate to poor patient survival in the TCGA data base, supporting further investigation.

While tumor cells in the OB and RH differ in terms of radiosensitivity, DSB repair and gene expression, they also show significant differences in neuronal contacts. Recent studies suggest that neurons contribute to glioma proliferation, invasion, and possibly treatment response [19, 35, 36]. The mechanisms have been attributed to GBM cells integrating into a neuronal electrical network via synapse-like structures and/or the secretion of paracrine factors [35, 37, 38]. Regarding the putative role of neurons in tumor behavior, the murine OB is a site of active neurogenesis and is highly enriched neurons and interneurons as compared to the cerebral hemisphere [25, 26]. Moreover, the OB was reported by Chen et al. to be a hotspot for tumor development in an autochthonous mouse model of gliomagenesis [38]. They showed that glioma development required neurosensory input from olfactory receptors and the release of neuronal IGF1, which then influenced tumor cells independently of synaptic formation. In the study reported here, because of the density of tumor-neuron somatic contact in the OB, synaptic like interactions between tumor cells and neurons were not specifically evaluated. However, in other areas of the murine brain as well as human brain, soma-soma contact between neurons and normal cells was shown to be indicative of cell–cell interactions, which were reflected in modifications in gene expression [20]. Accordingly, the increase in neuron/tumor cell contacts in OB versus RH (Fig. 7) suggest a greater frequency of cell–cell interactions and the increased opportunity of neurons to influence tumor cell behavior in the OB.

GBMs in the OB are relatively rare and there is no evidence indicating that their therapeutic response is different from those located in other regions of the brain. However, the significance of the radioresponse of human glioma cells in the murine OB, rather than as a direct comparison to the human OB, may be as a model system for investigating the mechanisms responsible for GBM radioresistance in situ. The response of a GBM to radiotherapy is determined by the most resistant subpopulation. The murine OB provides a source of radioresistant tumor cells not found in the standard GBM orthotopic xenograft models (i.e., cerebral hemisphere). Given its unique microenvironment, the murine OB may serve as an amplified version of a radioresistant niche operative on a considerably smaller scale in other areas of the brain, and thus more difficult to study. Along these lines, the murine OB offers a microenvironment enriched in neuron/tumor cell contact. Defining the mechanisms responsible for the radioresistance of tumor cells in the murine OB may thus provide novel insights into the molecules and processes mediating GBM radioresistance in situ.

## Conclusion

The radioresistance of GBM cells in the OB can be attributed to an increase in DSB repair, which is consistent with an increase in the expression of genes involved in NHEJ. Because the murine OB provides a radioresistant niche for GBM cells, it may serve as a model system for studying the mechanisms mediating GBM radioresistance and the development of radiosensitizing agents.

## Supplementary Information

**Additional file 1.** **Supplemental Methods**. Antibodies used for immunohistochemical analyses.

**Additional file 2.** **FigS1. Flow cytometry histograms**. A. Gating strategy: Gate 1 separates live cells from dead cells and debris; Gate 2 identifies GFP positive tumor cells. Doublets were excluded using two parameters (FSC-A x FSC-H and SSC-W x SSC-H). After gating, histograms were generated as cell number vs. DNA content (propidium iodide (PI) staining). B. Representative histograms showing the cell cycle distribution of NSC11 and NSC20 cells in the OB and RH as a function of time after 10 Gy. % cells in each cell cycle phase was determined using FlowJo software.

## Data Availability

All data generated or analyzed during this study are included in this published article or at NCBI’s Gene Expression Omnibus.

## Abbreviations

GBM: Glioblastoma
OB: Olfactory bulb
RH: Right hemisphere
DSB: Double strand break
SAC: Spindle assembly checkpoint
GSC: Glioblastoma stem-like cell
NHEJ: Non-homologous endjoining
CTCF: Corrected Total Cell Fluorescence
