# Supplementary material for: Glioblastoma cells have increased capacity to repair radiation-induced DNA damage after migration to the olfactory bulb
Source: Cancer Cell Int. 2022 Dec 8;22:389. doi: 10.1186/s12935-022-02819-0 (PMC9733339; doi:10.1186/s12935-022-02819-0)
Supplement: Supplementary file 1 — Additional file 1. Supplemental Methods. Antibodies used for immunohistochemical analyses. [file 12935_2022_2819_MOESM1_ESM.docx]

**Supplementary material and methods: Antibodies**

MAD2 (Sc374131) was obtained from SantaCruz. Bub1b (11504-2-AP) and TTK (10381-1-AP) were obtained from Invitrogen. Bub1 (ab195268), Cdc20 (ab1102), KU70 (ab202022), KU80 (ab119935), MRE11 (ab214), DNAPKcs (ab32566), CD90 (ab181469), CD133 (ab19898) CENPF (ab223847), MAP2 (ab183830) and TH (ab152) were obtained from abcam. Phospho-H3 (ser10) (FCMAB104A4) and Nestin (MAB5326) were obtained from Millipore. GABA (A2052) was obtained from Sigma and is produced in rabbit using GABA-BSA as the immunogen. The primary antibody incubation was done along with the human markers Sox2 (3579) or Sox2 (C761895) respectively obtained from Cell signaling and LSbio. Primary antibody incubation is then followed by Alexa fluor conjugated secondary antibodies from Invitrogen and 1ug/ml DAPI incubation for 1h at room temperature and mounted with Prolong Diamond antifade (Invitrogen).
