# Supplementary figures and images for: Glioblastoma cells have increased capacity to repair radiation-induced DNA damage after migration to the olfactory bulb
Source: Cancer Cell Int. 2022 Dec 8;22:389. doi: 10.1186/s12935-022-02819-0 (PMC9733339; doi:10.1186/s12935-022-02819-0)

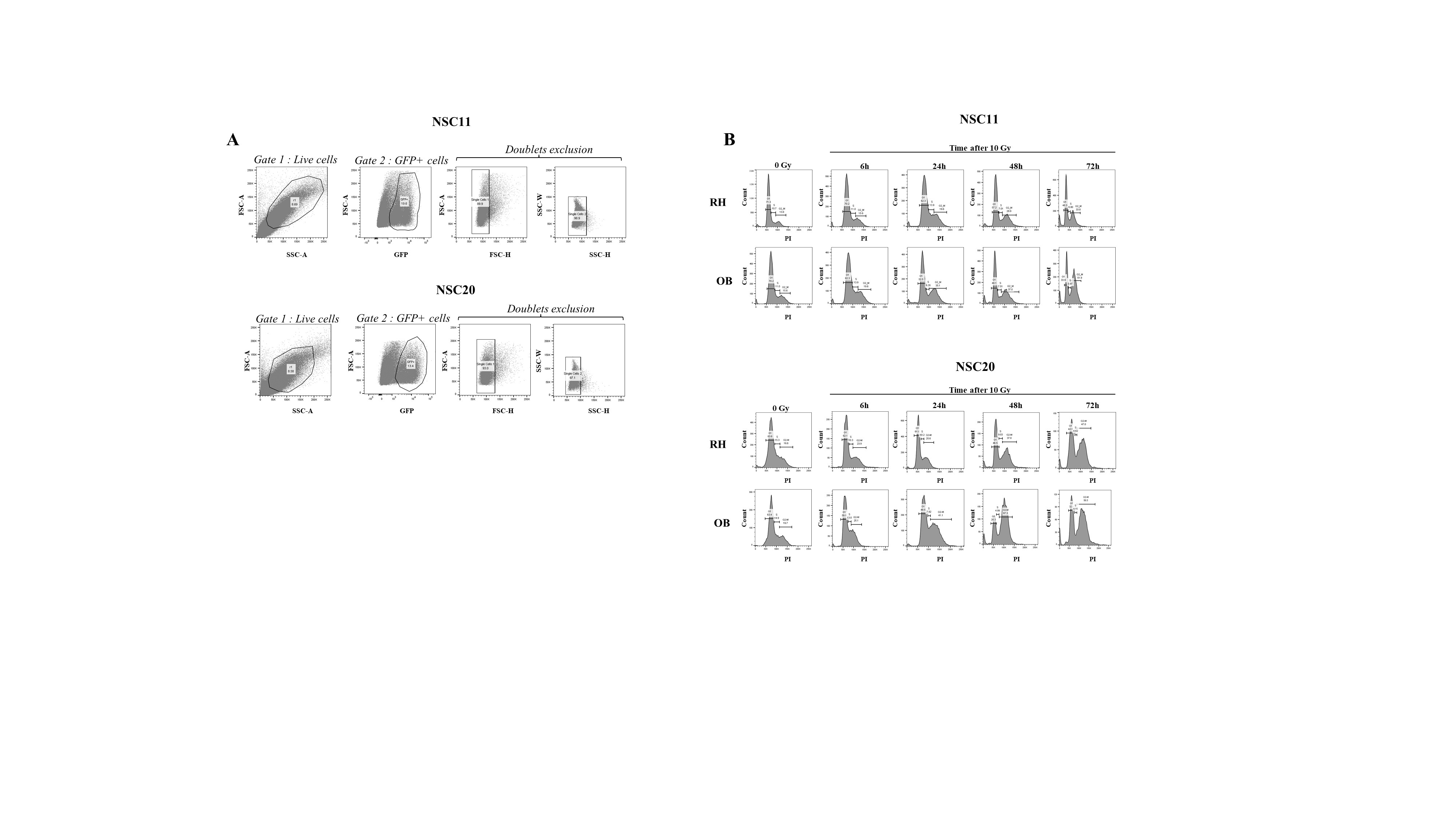

Supplement: Supplementary file 2 — Additional file 2. FigS1. Flow cytometry histograms. A. Gating strategy: Gate 1 separates live cells from dead cells and debris; Gate 2 identifies GFP positive tumor cells. Doublets were excluded using two parameters (FSC-A x FSC-H and SSC-W x SSC-H). After gating, histograms were generated as cell number vs. DNA content (propidium iodide (PI) staining). B. Representative histograms showing the cell cycle distribution of NSC11 and NSC20 cells in the OB and RH as a function of time after 10 Gy. % cells in each cell cycle phase was determined using FlowJo software. [file 12935_2022_2819_MOESM2_ESM.jpg]
